# Adjustment of family planning service statistics reports to support decision-making at central and governorate level, Egypt

**DOI:** 10.1186/s42506-021-00098-7

**Published:** 2022-01-20

**Authors:** Noha Asem Mohamed, Madiha Said Abdel-Razik, Marwa Rashad Salem

**Affiliations:** Department of Public Health and Community Medicine, Faculty of Medicine, Cairo University, Box 109 El Malek El Saleh, Cairo, PO 11559 Egypt

**Keywords:** Family planning, Management information system, Key performance indicators, Couple year protection, Contraceptive coverage rate, Egypt governorates, Egypt regions

## Abstract

**Background:**

The Ministry of Health and Population (MOHP)-Family Planning Sector (FPS) has a strong management information system (MIS) that allows the flow of data from MOHP-FP clinics, health districts, and governorates up to the central level. Yet, family planning (FP) quarterly reports issued at the central level are presented as database/spreadsheet software documents. These data are not used to provide indicators or information that aid in decision-making or the tracking of FP services over time. The objective of the study is to organize data in the database, develop key performance indicators, and design FP reports and policy briefs.

**Methods:**

The study is operations research that is driven by published data derived from MOHP-FP sector-head, and 2014 service statistics quarterly hardcopy reports. The information was entered into an excel program, and 15 key performance indicators (KPIs) were calculated and used to rank Egypt’s 27 governorates. We developed an annual FP report form, settled tables, and colored graphs that are liable to rank the governorates from best to least favorable.

**Results:**

The quarterly data sheets issued by the MOHP-FP sector were organized for the quarters, and one annual sheet was developed with the organization of Egypt’s Governorates into 4 specific regions, with each governorate having a fixed position in all reports. The key performance indicators were as follows: percent of clients aged 35 and up; percent of clients with fewer than three children; proportion of current FP users by method; percent of clients reported as first-time clients; percent of clients defined as new clients (non-FP users and FP discontinuers); and contraceptive coverage rate, i.e., percent coverage of married women of reproductive age with dispensed FP methods expressed as couple years.

**Conclusion:**

MOHP-FP sector service statistics data could be used for the development of fifteen key performance indicators. Having those indicators at governorate, district, and central levels in quarterly and annual reports and their communication with decision-makers at all levels and their tracking overtime will guide them to timely decision-making for improving performance in FP services at all levels.

## Introduction

In Egypt, the drop in fertility rates has slowed since the beginning of the millennium. As of 2006, an increased fertility trend has been seen, peaking in 2014 before decreasing again in 2015, with an accelerated pace in 2018 [1, 2].

Egypt has adopted family planning as a short-term strategy to reduce the population growth rate since the early sixties. The set goal of national population policy (NPP) in Egypt is to reduce the population growth rate and to achieve replacement level fertility, with a total fertility rate of 2.1 children per woman. To achieve the goal of the NPP, strategic objectives included the increase in the contraceptive prevalence rate (CPR) of 73% [3].

Family planning services are provided through a wide network of public (governmental) and private (non-governmental) facilities. Public facilities are mostly run by the MOHP and include primary health care facilities and hospitals, while private facilities include for-profit (private doctors, private pharmacies, and private hospitals), and non-governmental organizations (NGOs). The public sector, namely MOHP, is the largest provider of FP services in Egypt, used by 57% of FP users [2]. There are nearly 6000 FP clinics affiliated with MOHP, including rural health units, urban health centers, family health units, clinics at general hospitals, and mobile clinics. MOHP facilities offer FP services at nominal fees **[**4].

At the program level, high-quality data is essential for evidence-based decision-making to support successful FP programs. Consistent recording and reporting practices, optimal access to and use of health information systems, and rigorous interpretation and use of data for decision-making are characteristics of high-quality information systems [5].

On the demand side, not all methods are favored at the same time. This is influenced by differences in the range of contraceptive technologies’ availability, accessibility, and acceptability [5, 6]. Accordingly, there is an increasing importance of monitoring trends and determinants of method choice as family planning and reproductive health programs must adapt to meet users’ changing needs and preferences [7]. Access and service quality are important factors for contraceptive acceptance and continuation [8].

According to the National Population Council (NPC), the contraceptive coverage rate (CCR) is used as a proxy indicator for monitoring the contraceptive prevalence rate (CPR). The CCR is the proportion of distributed FP methods (expressed as couple year protection/CYP) to the total number of married women in child-bearing age. According to NPC statistics, CCR had shown a decrease from 58% in 2005 to 53% in 2010 [9, 10].

In Egypt, it was discovered that the increase in the “all method” discontinuation rate from 2008 to 2014 was mostly due to the shift away from IUDs and toward hormonal treatments, which have higher discontinuation rates [11].

The Ministry of Health and Population (MOHP)-Family Planning Sector (FPS) has a management information system (MIS) that allows the flow of MOHP-FP clinics’ data from the health facility to the district, governorates, and central level. The quarterly reports and annual reports were designed and published by collaborative efforts of MOHP staff members and technical assistance during the donor USAD-supported project of the MOHP System Development Project (1990–2005). The MOHP-FP sector continued using the same FP form up to 2021. The FP quarterly reports continued to be published in hard copies up to the year 2014. However, after 2014, the same report forms were completed and kept for internal use only in the MOHP-FP sector.

Although the MOHP-FP sector has valuable MIS at the health facility level up to the central level, FP reports issued at the central level are presented as database/spread sheets hardware documents for the number of clients by age, parity, the FP method used, and services provided to the clients for each of Egypt’s 27 governorates. The data flow of FP-MIS indicated that electronic recording of data starts at the district level, then moves to the governorate level, finally reaching the central level. Yet, there is no processing of data to identify strengths and shortcomings across governorates for the allocation of resources and technical assistance. There is no feedback of information to peripheral levels to promote their role in action-taking. Consequently, there is no utilization of FP service statistics (FPSS) to develop key performance indicators (KPIs) for policy and strategy development, design operational plans based on the current situation, and monitor KPIs over time and across governorates. The current study was conducted to revise the MOHP-FP quarterly statistical reports for Egypt’s 27 governorates (2014), set a list of KPIs for MOHP-FP at the governorate level, and upgrade the performance report to guide decision-making and allocate resources, efforts, and improve efficiency according to prioritization of needs at the governorate level.

## Methods

### Study design

This is an analytic cross-sectional study that was performed on FP service statistics (SS) (2014) available in the MOHP-FP sector. The SS for the year 2014 was selected as it is the last hard copy of a quarterly report permitted for publication/use by researchers, as per a regulation set by MOHP-FP sector. Additionally, having MOHP-FP data allows for articulation of information with the last EDHS (2014) published in Egypt. Both input and output indicators were designed and comparisons between governorates were made. Accordingly, performance indicators were used to track the progress towards achieving FP program goals and objectives at the national level. Additionally, input and output indicators were ranked across governorates to identify those that need resources and/or technical assistance.

### Sources of data and data collection

The MOHP-FP Sector Headquarter was the source of the data. The authors received a hard copy of the report. The MOHP-FP sector Management Information System (MIS) tracks FP service activities through the flow of data from the FP clinic up to the central level. FP data is recorded at FP clinics and hard copies need to be sent to the FP unit in the Health District (each district manages a group of FP clinics). The FP Health district unit is responsible for data entry on special computer sheets. The completed electronic sheets have to be sent to the Health Directorate at the governmental level to compile all districts’ data into one sheet that presents FP data at the governorate level. The Governorates’ electronic FP forms are received at MOHP-FP Sector-Head Quarters to develop a final sheet that covers all Egypt Governorates. This final sheet is issued as a quarterly report. The recoding of each governorate in the final MIS reports is organized according to the time of receiving the governorate report. Therefore, each governorate has different positions in the quarterly reports, making it difficult to track each governorate over time. All the recorded data at all levels is presented as counts or numbers rather than proportions of indicators.

The service statistics reports at all levels entail 25 variables. The variables are: age of the client (5 groups), number of children (5 groups), currently used FP method (6 groups), type of the visit (two groups), type of the client (2 groups), reason of the visit (4 groups), and CCR indicator.

### Statistical evaluation

The hard copy data was entered into the computer Excel sheets. Reorganization of data in the excel sheet was done so as to have logic and constant well-organized groups of 27 governorates into urban governorates (4 governorates), lower Egypt governorates (9 governorates), upper Egypt governorates (9 governorates) and frontier governorates (5 governorates). Key performance indicators (KPIs) were calculated and used for rank ordering of Egypt 27 governorates. The number of married women in the reproductive age (MWRA) at Governorate level was derived from the National Population Council–Statistical annual report for 2014. The hard copy of the report presented data as counts/numbers. Analysis included using percentages across data at governorate and national level. Rank ordering of governorates according to specific indicators related to service delivery output indicators was done. Using of centiles allowed for categorization of governorates into three groups: best situation (green color), unfavorable situation (red color), and prospective situation (yellow color).

### Definition of key performance indicators in FP services

1. Percent share of each of Egypt governorates in the total married women in the reproductive age in Egypt

- This indicator presents the volume of target women for FP services at governorate level in relation to the national target women for FP program.
- The higher the percent of the target volume the needs for resources and their proper management to improve efficiency (output of FP services in the FP clinic). However, with stability of the community, this indicator will remain constant over long time.

(2) Percent of MOHP-FP clinics in certain governorate to total MOHP-FP clinics at the national level

- This is an input indicator that shows the present distribution of health services resources across the country, i.e., MOHP-FP clinics.
- For each governorate, this indicator can be considered with the other indicators as a percent of MWRA. The lower the value of this indicator for a certain governorate in relation to other governorates that have the same proportionate target of percent of MWRA, the more decisions for increasing service delivery points in those governorates could be made.

(3) The number of MOHP-FP clinics on average
(4) Rate of contraceptive coverage

- This indicator is usually used by the MOHP-FP sector and the National Population Council. It is a proxy indicator for the contraceptive prevalence rate.
- It is the percentage of MWRA within the catchment area of the health facility who are protected from pregnancy for 1 year due to FP methods dispensed by the FP clinic, expressed as couple years of protection (CYP).

CYP expresses contraceptive methods dispensed as the number of couples served by family planning methods for 1 year. An example: if a family planning clinic has dispensed four injectable vials, the dispensed injectables are equivalent to one CYP. In the case of dispensing 8 injectables, the CYP is equivalent to two CYP (or two couples are protected from pregnancy for 1 year) (Table 1).

**Table 1:** Calculation of FP couple years of protection

Contraceptive methods dispensed	Conversion factor (in Egypt)	Description
Pills (cycles)	Divide by 13	13 cycles per couple/year
Condoms (units)	Divide by 100	100 units per couple/year
IUD (units)	Multiply by 3.2	Provides 3.2 years of protection on the average
Three monthly Injectable	Divide by 4	4 units per couple/year
Monthly injectable	Divide by 13	13 cycles per couple/year
Implanon	Multiply by 2	Provides 2 years of protection on the average
Sterilization (procedure)	Multiply by 10	Provides 10 years of protection on the average

$$ \mathrm{CCR}=\frac{\mathrm{CYP}\ \mathrm{for}\ \mathrm{dispensed}\ \mathrm{FP}\ \mathrm{methods}\ \mathrm{by}\ \mathrm{the}\ \mathrm{FP}\ \mathrm{Clinic}\ \mathrm{in}\ \mathrm{a}\ \mathrm{certain}\ \mathrm{period}\ \mathrm{of}\ \mathrm{time}}{\mathrm{MWRA}\ \mathrm{within}\ \mathrm{the}\ \mathrm{catchment}\ \mathrm{area}\ \mathrm{of}\ \mathrm{the}\ \mathrm{FP}\ \mathrm{clinic}\ \mathrm{in}\ \mathrm{the}\ \mathrm{same}\ \mathrm{time}\ \mathrm{period}}\times 100 $$

In the current study, this indicator is calculated by the MOHP-FP sector for contraceptive methods dispensed to clients in 2014. Those methods are dispensable for three categories of clients:

- Those who are current FP users and sought an FP clinic to get the method for continuation of use
- New users of the FP method
- Clients attended the FP clinic to change the contraceptive method.
- Higher values of this indicator indicate that FP clinics dispense either a high number of units of contraceptive methods or a small number of contraceptive units that have a higher conversion factor.
- This indicator could be measured at the FP clinic level, district level, governorate level, and national level.

(5) Percent distribution of FP clients by age category

FP clients are categorized into five age groups: < 20, 20–24, 25–29, 30–34, and 35 and more years. High proportion of FP-clients who are in the age group less than 35 years old indicates success of FP clinics in attracting young women for birth spacing or limiting.

(6) Percent distribution of FP clients by number of children

FP clients are categorized into five groups according to the number of children: none, one, two, three, four, and more children. The high proportion of FP clients who reported having less than three children indicates the success of FP clinics in attracting low parity women for birth spacing or limiting.

(7) Percent distribution of FP clients by type of client (new versus continuing) by governorate

A new client is defined as a client who has never used the FP method before or has discontinued using contraceptive methods for 1 year or more before attending the FP clinic. The higher the proportion of new clients versus continuing clients, the more efficient FP clinics are in attracting new FP clients.

(8) Percent distribution of FP clients by type of visit (1st visit versus return visit) by governorate

Clients who attend MOHP-FP clinic for the first time are recorded in MOHP service statistics as first visit. The higher the proportion of first visit clients versus return visits indicates the efficiency of MOHP-FP clinics in motivating newcomers. The client who attends as first visit could be utilizer of other FP clinics and could be user or non-user of FP method.

(9) Percent distribution of FP clients by reasons of the visit (get FP method, counseling, RH services, and management of FP method side effects) by governorate

The percent distribution of clients according to reasons of visits to FP clinics is a good indicator as it included other 4 indicators. Higher proportion of clients attending FP to get FP methods indicates the role of FP clinics supplying FP users by methods and ensure continuation of use. Additionally, it reflects the capability of FP clinics of having good cafeteria of satisfactory contraceptive method mix.

(10) Percent distribution of FP clients by current contraceptive method used by governorate

This indicator describes the FP clients currently using FP method by contraceptive method mix. This indicator could be expressed as 6 indicators according to FP methods currently used by clients. This is a proxy indicator for the type of service that clients seek from FP clients. Users of oral contraceptives, condoms, three monthly and one monthly injection, usually seek the FP clinics mainly for restock/replenish FP methods. Users of IUD and implants could attend FP clinics for management of side effects and reproductive health services or for changing the method.

## Results

Table 2 illustrates the total number of married women in reproductive age (MWRA), and percent distribution across governorate. The same table shows the total number of MOHP-FP clinics and their percent distribution among Egypt governess and average number of FP clinics per 10,000 MWRA. The output of MOHP-FP clinics throughout 2014 and expressed as contraceptive coverage rate is presented by Egypt Region and corresponding governorates. The total number of MWRA in Egypt 2014. The total number of MWRA was more than 15 million MWRA (*n* = 15799805). MWRA was distrusted in Egypt regions with higher proportion in lower Egypt (44%) and upper Egypt (38%). The total number of MOHP-FP clinics were 6038 clinics. About half of those clinics (49%) serve upper Egypt governorates, and 47% serve lower Egypt governorates. The average number of clinics per 10,000 MWRA was 3.8 MOHP-FP clinics. This ratio was minimum at urban governorates (2.6) and maximum at the frontier governorates (14.8). The output of 6038 MOHP-FP clinics in 2014, expressed as percent coverage of MWRA in Egypt with contraceptive methods for 1 year, i.e., percent of CCR was 37%. Lower Egypt governorates reported higher achievement in CCR (42%), followed urban governorates (32%).

**Table 2:** Distribution of married women in the reproductive age, MOHP-FP clinics and contraceptive coverage rate across Egypt governorate 2014

Governorates	%MWRA	% MOHP FP clinics	MOHP FP clinics/10,000 MWRA	CCR %
Cairo	10	4.7	1.8	33
Alexandria	5	3.1	2.2	33
Port-Said	1	0.6	3	30
Suez	1	0.6	3.2	31
**Urban G**	**17**	**9**	**2.6**	**32**
Damietta	2	1.8	4.4	32
Kafr-Elshieikh	4	4.7	5	36
Gharbia	5	5.3	3.8	43
Dakahlia	7	9	5	46
Qalubia	5	3.6	2.3	40
Menofia	5	4.5	3.8	41
Behera	7	7.9	4.7	57
Ismailia	1	1.3	3.7	45
Sharkia	8	8.6	4.3	40
**Lower Egypt**	**44**	**46.7**	**4.1**	**42.2**
Aswan	2	3.9	9.6	26
Giza	9	4.5	1.9	36
Luxor	1	2	5.9	24
Quena	3	4.4	5	19
Fayoum	4	3	3	40
Beni-Suef	3	3.5	4	43
Souhag	5	6.2	4.5	19
Mynia	6	6.7	4.4	47
Asuit	5	4.8	3.9	28
**Upper Egypt**	**38**	**39.0**	**4.7**	**31.3**
Red Sea	0.4	0.7	6.7	25
New Valley	0.3	1.1	16.4	37
Matrouh	0.4	1.2	10.4	26
N Saini	0.5	1.6	12.5	17
S. Saini	o.1	0.8	28.2	23
**Frontier Governorates**	**1.6**	**5.4**	**14.84**	**25.6**
Total Egypt	15,799,805	6038	3.8	37

Bold refers to the geographical classificataion of governorates in Egypt

**Fig. 1:**
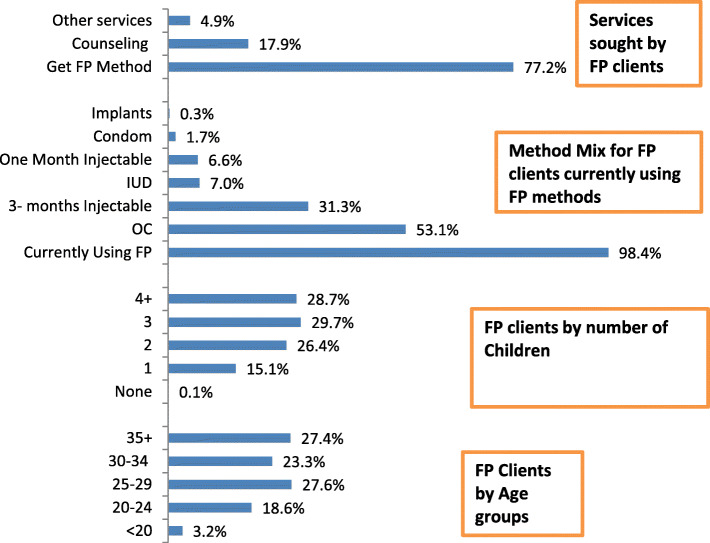
Profile of MOHP- FP clinics’ clients (age and number of children), who were currently using contraceptive method, reasons of visits to FP clinics in 2014 (total clients = 13,846,714)

Figure 1 summarizes findings of the estimated indicators for 13,846,714 clients attended MOHP-FP clinics in 2014. FP clients who were in the age group 35 and more years formed 27.4% and those who were less than 20 years old formed 3.2%. Indicators that reflect parity profile of FP clients showed that those who have three children and more formed 41.6%. Out of the total FP clinics’ attendance 98.4% were already current users of FP methods. More than half of the clients were using oral contraceptives (OC) (53.1% and about one third (31.3%) were using three-month injectables. The major reason for seeking services in MOHP-FP clinics was getting contraceptive methods (77.2%).

Table 3 presents the situation of current FP method use among MOHP-FP clinics’ client and distribution of FP method users by type of FP method across Egypt. More than half (54%) were OC users and about one third (31%) of clients were 3-month injectable users. However, there was a tendency for frontier governorates to be OC users (64%).

**Table 3:** Distribution MOHP-FP clinics’ clients who were currently using contraceptive methods by type of the method, governorate and region, Egypt, 2014

Governorates	Percent of MOHP-FP clinics’ clients according to current FP method used						
	% OC users	% IUD users	%Condom users	Three-month injectable	Implants	Monthly injectable	Total clients using FP method
Cairo	42	14	7	29	0.5	3.5	708,152
Alexandria	35	14	8	35	0.4	2.4	361,784
Port-Said	36	9	12	42	0.3	0	77,908
Suez	54	9	3	25	0.6	0	71,036
**Urban Governorates**	**39**	**17**	**7**	**34**	**0.6**	**2.8**	**1,218,880**
Damietta	61	6	4	22	0.2	0.0	207,108
Kafr-Elshieikh	47	6	1	39	0.2	0.0	492,016
Gharbia	57	9	2	24	0.2	0.0	647,728
Dakahlia	48	9	2	34	0.2	0.0	978,268
Qalubia	50	8	2	33	0.2	0.0	772,504
Menofeya	62	5	1	24	0.1	4.4	813,220
Behera	47	9	1	32	0.1	0.0	1,098,520
Ismailia	48	6	2	36	0.1	0.0	206,468
Sharkia	66	3	1	23	0.1	5.4	1,699,280
**Lower Egypt**	**55**	**9**	**1.8**	**33**	**0.2**	**1.9**	**6,915,112**
Aswan	75	2	1	16	0.3	0.0	286,260
Giza	56	10	1	28	0.1	0.2	998,424
Luxor	79	1	1	12	0.3	0.0	300,964
Quena	73	1	1	25	0.3	0.0	516,988
Fayoum	37	6	1	51	0.4	2.9	578,980
Beni-Suef	42	7	0.5	41	0.2	4.8	498,612
Souhag	59	3	1	31	0.2	0.7	553,064
Minia	48	4	1	42	0.2	0.8	1,096,052
Asuit	54	5	1	32	0.2	5.6	529,608
**Upper Egypt**	**54**	**7**	**0.9**	**36**	**0.2**	**1.6**	**5,358,952**
Red Sea	70	4	3	16	0.3	0	44,420
New Valley	68	5	2	20	0.4	0	41,108
Matrouh	43	5	2	48	0.6	0	43,336
N Saini	62	5	0.5	32	0.6	0	31,724
S. Saini	75	2	3	20	1.1	0	16,572
**Frontier Governorates**	**64**	**6**	**2.1**	**25**	**0.3**	**0**	**177,160**
Total MOHP-FP Clients using FP methods	7,376,884	903,636	255,016	4,228,168	27,388	879,012	13,670,104
%	54	7	2	31	0.2	6	100

*OC* oral contraceptives, *IUD* intra-uterine contraceptive device

Bold refers to the geographical classificataion of governorates in Egypt

Table 4 shows the distribution of MOHP-FP clinics’ clients according to type of the visit, type of the client and reasons for seeking FP clinics’ services. One fifth (21%) of the clients were recorded as first visit to MOHP-FP clinic. However, urban governorates recorded higher figure for the percent of first visit clients (34%) compared to other Egypt regions. New clients as non-FP users before or discontinuers of FP methods for 1 year and more formed 11% of all MOHP-FP clinics’ clients. Urban Governorates have recoded a figure of 14% for the new client indicator. The major cause of seeking MOHP-FP clinic’ services was getting FP method, either for new clients or for the current users (83%) with a range across regions from 79% for lower Egypt governorates to 83% for frontier governorates. Counseling services were sought by 14.4% of MOHP-FP clinics’ clients, with a range of 8.8% in urban governorates to 15.9% in lower Egypt governorates.

**Table 4:** Distribution of MOHP-FP clients according to type of the visit, type of client, and reasons for seeking services at FP clinics, by governorates, 2014 Egypt

Governorates	Type of visit	Type of client	Reasons for seeking MOHP-FP clinics by FP clients			
	% of 1st visit clients	% of new clients	% get FP method	% Counseling services	% RH services	% management of contraceptive methods’ side effects
Cairo	34	15	81	7.5	11.3	18.9
Alexandria	35	15	85	3.1	11.5	14.6
Port-Said	28	10	71	22.2	6.8	28.9
Suez	33	10	67	27.1	6.3	33.4
**Urban Governorates**	**34**	**14**	**81**	**8.8**	**10.7**	**19.5**
Damietta	18	8	88	2.9	8.8	22.2
Kafr-Elshieikh	19	9	84	10.9	4.7	35.5
Gharbia	19	10	64	27.2	8.3	38.1
Dakahlia	23	11	78	18.2	4.0	18.3
Qalubia	22	11	92	3.4	5.1	15.6
Menofeya	15	7	62	35.4	2.7	6.3
Behera	22	9	94	0.2	6.0	25.9
Ismailia	22	9	74	17.8	8.1	21.3
Sharkia	16	8	82	12.7	5.6	8.5
**Lower Egypt**	**19**	**9**	**79**	**15.9**	**5.4**	**21.3**
Aswan	17	9	81	15.7	3.8	18.4
Giza	28	14	81	12.3	6.9	12.6
Luxor	13	5	82	14.6	3.7	19.5
Quena	14	9	96	0.3	3.6	18.3
Fayoum	23	13	55	42.0	3.0	3.4
Beni-Suef	28	15	87	4.8	7.8	45.0
Souhag	23	16	75	20.1	4.9	3.9
Minia	23	12	97	0.9	2.5	8.0
Asuit	16	12	92	3.2	4.8	25.0
**Upper Egypt**	**22**	**12**	**82**	**13.8**	**4.5**	**18.4**
Red Sea	18	5	85	8.4	6.4	14.7
New Valley	14	6	69	27.9	3.6	31.4
Matrouh	33	20	97	0.1	3.4	3.5
N Saini	23	21	94	1.1	4.9	6.1
S. Saini	20	12	76	11.8	12.1	23.9
**Frontier Governorates**	22	13	83	11.4	5.3	16.7
Total MOHP-FP Clinics Clients	21	11	83	14.4	5.6	19.9

Bold refers to the geographical classificataion of governorates in Egypt

**Fig. 2:**
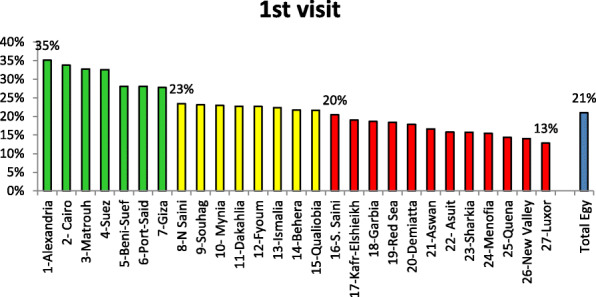
Rank order of MOHP-FP clinics at governorate level according to the percent of clients’ reported first visit to FP clinics

Figure 2 illustrates a simple indicator for measuring capability of MOHP-FP clinics to attract clients to utilize FP clinics for the first time. Alexandria governorate occupied the first position regarding higher percentage of FP clients who report first visit to the MOHP-FP clinic. Grouping governorates according to this indicator revealed best situation group, i.e., located within a range of 35%—more than 23%. Governorate with unsatisfactory situation revealed an indicator value from 20 to 13%.

**Fig. 3:**
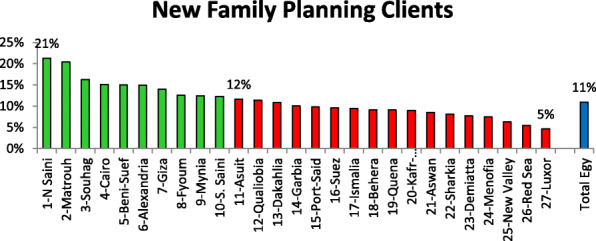
Rank order of MOHP-FP clinics at governorate level according to the percent of clients reported as new FP clients (non-FP users, and discontinuers of FP use for 1 year and more)

Figure 3 elucidates the indicator reflecting competence of MOHP-FP clinics to attract clients to start (first time) and/or regain (reuse after discontinuation) the use of FP method. Rank order is illustrated for Egypt Governorates from the best situation to unfavorable situation according to percent of new FP clients to total clients. There were 10 governorates who were categorized as best situation governorates according to this new FP client’s indicator. The other governorates (*n* = 17) were categorized as unfavorable situation.

**Fig. 4:**
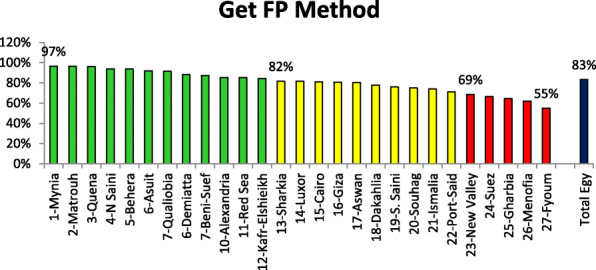
Rank order of MOHP-FP clinics at governorate level according to the percent of clients reported attendance to FP clinics to get FP methods

Figure 4 reveals the indicator that assesses the capability of MOHP-FP clinics to have and dispense FP methods to clients who are new clients as well as replenish FP method to current FP method users. This indicator was termed as “the reason for attending FP clinic for getting FP methods”. Rank order is demonstrated for Egypt governorates from the best situation to unfavorable situation according to percent of FP clients attending FP clinic to get FP methods to total clients. Minia Governorate ranked the first (97%) regarding this indicator. There were 12 governorates who were categorized as best situation governorates according to the indicator of “attending FP clinic to get FP methods. Governorates were categorized as having unfavorable situation were 5 governorates.

**Fig. 5:**
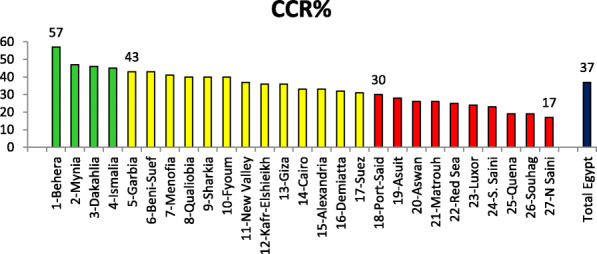
Rank order of MOHP-FP clinics at governorate level according to the percent of coverage of MWRA by FP methods in 2014

Figure 5 demonstrates an important indicator that link between performance of the MOHP-FP clinics in coverage of the target MWRA at community level, i.e., contraceptive coverage rate (%CCR). Ranking of Egypt governorate according to this indicator showed that Behera Governorate ranked number 1. There were 4 governorates categorized as best situation governorates, and 10 governorates located in the unfavorable situation group.

## Discussion

This study is concerned with family planning service provided by the public/governmental sector through MOHP-FP clinics in Egypt. This public sector was the source of FP methods for 56.7% of contraceptive method users [2]. While other studies used FP service statistics were aiming at assessment of the capability of service statistics in annual tracking of contraceptive prevalence rate (CPR), which is a population/community-based outcome indicator [12, 13], we aimed in this study to generalize the methodology of adjusting MIS reports to inform policy makers at all levels about the performance of FP clinics.

The study raised the initiative of designing the format for recording data at all levels to provide essential data and reduce redundancy. Consequently, the study identified 15 variables and developed 15 key performance indicators for each governorate, Egypt regions, and the national levels rather than having 25 variables for each governorate.

The study worked on the final report issued by the MOHP-FP Sector MIS. The first step is to organize Governorates with reasoning arrangement according to Egypt regions. As this region-oriented arrangement reflects the background of the governorate that could explain findings from service statistics. Urban governorates include urban cities, with Cairo and Alexandria are big cities. Urban governorates are privileged governorates due to easy access to all health and educational services. Lower Egypt governorates have rural communities that prefer large families for economic reasons. Upper Egypt governorates are characterized by having rural as well as conservative communities. Frontier governorates are inhabited with trips with conservative culture and live in a wide space land. Therefore, we could compare governorates regarding FP clinics’ utilization within the same region. The arrangement of governorate with a fixed location in all MIS reports could allow merging of quarterly report data to develop annual reports.

The study provided important information about the target group, i.e., MWRA for FP program. This target was different across governorates. Considering the average number of MOHP-FP clinics per 10,000 MWRA is an important indicator for assessment of MOHP resources across governorates, in decisions for allocating more resources as service delivery points such as mobile clinics and mobile teams to governorate having shortage in number of FP clinics. Data on this indicator are not available in the FP-MIS reports.

The MOHP-FP quarterly reports include variables related to the age of clients categorized into 5 groups that could provide 5 indicators to describe the proportionate distribution of the clients by age group. The current study focused on only one indicator which is the proportion of clients 35 and more years old. This indicator demarcates one important priority categories for FP services. Thus, high value of this indicator in a certain governorate in relation to other governorates is more likely to be interpreted as this high-risk group needs more effort from FP clinics for management of comorbidities in addition to using FP methods that is appropriate for each client in the age 35–49 years.

Focusing on the proportion of clients who have 3 and more children is crucial for FP where efforts are directed towards spacing and limiting. This group includes also multiparous women who are at the risk of reproductive health problems and medical problems due to repeated pregnancy. Although the serious health problems usually start after the fourth child, it is necessary to prepare women for FP use after the third child [14].

The percent distribution of MOHP-FP clients by the currently used contraceptive methods is an important guide for MOHP contraceptive security issues [15]. Those using OC attend FP clinic for replenishing the packets on monthly basis. Shortage in availability of specific method could lead to discontinuation of contraceptive method. This problem is obvious in case of the one-monthly injectable and implants. The contraceptive security and procurement departments at MOHP-FP sector could benefit from such indicator to estimate the needed amount for different contraceptive methods. This issue is related to the supply component of FP system.

The demand component of the FP system entails the capability of FP clinics (which are usually located in primary health care units and centers as well as family medicine units and center) to communicate with other clinics in health units and centers to refer women to get FP services in the corresponding FP clinic [16]. Additionally, community workers play a role through home-visit to motivate women for accepting visits to MOHP-FP clinics. Community workers who do good job, are those who motivate and refer women to pay the first visit to FP clinic, and motivate non-users, especially discontinuers and those who did not use FP method before, with subsequent increase in the proportion of FP clients recorded as first visit client as well as new client who did not use FP method before and/or FP discontinuers for 1 year and more.

The reasons of visiting MOHP-FP clinics included 4 indicators. The proportion of FP clients attended to get FP methods has high implication. It is obvious that if high proportion of FP clients visit FP clinics for just getting the method, especially for OC, injectables, and condoms, this may reflect the situation that the low price, and lack of need for clinical examination or gynecological examination, are the insistent factors to visit FP clinics, which work in this condition as a pharmacy for dispensing FP methods for clients of recurrent visits.

The study provided valuable models of info-graphs for development of policy briefs to policy makers at all levels. The colored charts for Egypt governorates are presented in rank order from best situation (green color), and unfavorable situation (red color) and probable o change into unfavorable situation (yellow color). The four indicators have important implications. The first and second indicators are as follows: (1) the proportion of clients recorded as first visit and (2) the proportion of clients recorded as new clients are concerned with raising demands for FP services. Governorates in the red region need to train community workers to guide women to visit FP clinics. The third indicator is (3) the proportion of clients attended FP clinic to get FP methods draws the attention of the governorates located in the red zone to be supported by the contraceptive security department at MOHP-FP sector. The fourth indicator is (4) contraceptive Coverage Rate is an indicator that link contraceptive methods dispensed by MOHP-FP clinics (expressed as couple year protection) per 100 MWRA. The more is the dispensing of FP methods especially IUD and implants (have high conversion factor) the more is the value of this indicator. This indicator includes methods dispensed to current users and new users. In other words, and according to annual FP report this indicator reflect the percent of MWRA protected from pregnancy for 1 year.

For the development of policy brief at the central, governorate, and district levels, the “Title” should be added and phrased as suggested recommendation as for example, demand creation through training of community is pivotal to increase FP new users in Souhag Governorate. The “Introduction” section includes topic importance, objectives, methodology, sources of data, and who conducted data management. The “Major Results” section includes specific results that guide for recommendations. The “Recommendation” section should be derived from the key findings [17].

This study is concerned with family planning services provided by the public or governmental sector through MOHP-FP clinics in Egypt. This public sector was the source of FP methods for 56.7% of contraceptive method users [2]. While other studies using FP service statistics were aimed at an assessment of the capability of service statistics in annual tracking of contraceptive prevalence rate (CPR), which is a population/community-based outcome indicator [12, 13], we aimed in this study to generalize the methodology of adjusting MIS reports to inform policy makers at all levels about the performance of FP clinics.

The study also raised the issue of designing the format for recording data at all levels to provide essential data and reduce redundancy. Consequently, the study identified 15 variables and developed 15 key performance indicators for each governorate, Egypt’s regions, and the national levels, rather than having 25 variables for each governorate.

The study worked on the final report issued by the MOHP-FP Sector MIS. The first step is to organize governorates with a reasonable arrangement according to Egypt’s regions. As this region-oriented arrangement reflects the background of the governorate, that could explain findings from service statistics. Urban governorates include urban cities, with Cairo and Alexandria being big cities. Urban governorates are privileged governorates due to easy access to all health and educational services. For economic reasons, the lower Egypt governorates have rural communities that prefer large families. Upper Egypt governorates are characterized by having rural as well as conservative communities. Frontier governorates are populated by people who practice conservative culture and live over a large area of land. Therefore, we could compare governorates regarding FP clinics’ utilization within the same region. The arrangement of governorates with a fixed location in all MIS reports could allow the merging of quarterly report data to develop annual reports.

The study provided important information about the target group, i.e., MWRA for the FP program. This target was different across governorates. Considering the average number of MOHP-FP clinics per 10,000 MWRA is an important indicator for the assessment of MOHP resources across governorates, in decisions for allocating more resources as service delivery points such as mobile clinics and mobile teams to governorates having a shortage in the number of FP clinics. Data on this indicator is not available in the FP-MIS reports.

The MOHP-FP quarterly reports include variables related to the age of clients categorized into 5 groups that could provide 5 indicators to describe the proportionate distribution of the clients by age group. The current study focused on only one indicator, which is the proportion of clients aged 35 and older. This indicator demarcates one important priority category for FP services. Thus, the high value of this indicator in a certain governorate in relation to other governorates is more likely to be interpreted as this high-risk group needing more effort from FP clinics for management of comorbidities in addition to using FP methods that are appropriate for each client aged 35–49 years.

Focusing on the proportion of clients who have 3 or more children is crucial for FP where efforts are directed towards spacing andlimiting. This group also includes multiparous women who are at the risk of reproductive health problems and medical problems due to repeated pregnancy. Although the serious health problems usually start after the fourth child, it is necessary to prepare women for FP use after the third child [14].

The percent distribution of MOHP-FP clients by the currently used contraceptive methods is an important guide for MOHP contraceptive security issues [15].Those using OC attend an FP clinic to replenish the packets on a monthly basis. A shortage in the availability of specific methods could lead to the discontinuation of contraceptive methods. This problem is obvious in the case of the 1-monthly injectable and implants. The contraceptive security and procurement departments in the MOHP-FP sector could benefit from such an indicator to estimate the needed amount for different contraceptive methods. This issue is related to the supply component of the FP system.

The demand component of the FP system entails the capability of FP clinics (which are usually located in primary health care units and centers as well as family medicine units and centers) to communicate with other clinics in health units and centers to refer women to get FP services in the corresponding FP [16]. Additionally, community workers play a role through home-visits to motivate women to accept visits to MOHP-FP clinics. Community workers who do a good job are those who motivate and refer women to pay for their first visit to the FP clinic and motivate non-users, especially discontinuers and those who have not used the FP method before, with a subsequent increase in the proportion of FP clients recorded as first-time clients as well as new clients who have not used the FP method before and/or FP discontinuers for 1 year or more.

The reasons for visiting MOHP-FP clinics included four indicators. The proportion of FP clients who attend to get FP methods has high implications. It is obvious that if a high proportion of FP clients visit FP clinics for just getting the method, especially for OC, injectables, and condoms, this may reflect the situation that the low price and lack of need for clinical examination or gynecological examination are the primary factors for visiting FP clinics, which work in this condition as a pharmacy for dispensing FP methods to clients on recurrent visits.

The study provided valuable models of info-graphs for the development of policy briefs for policymakers at all levels. The colored charts for Egypt’s governorates are presented in rank order from best situation (green color), to unfavorable situation (red color), and probable situation (yellow color). The four indicators have important implications. The first and second indicators, which are (1) the proportion of clients recorded as first visits and (2) the proportion of clients recorded as new clients, are concerned with raising demand for FP services. Governorates in the red region need to train community workers to guide women to visit FP clinics. The third indicator (3), the proportion of clients who attended the FP clinic to get FP methods, draws the attention of the governorates located in the red zone to be supported by the contraceptive security department at MOHP-FP sector. The fourth indicator, (4) contraceptive coverage rate, is an indicator that links contraceptive methods dispensed by MOHP-FP clinics (expressed as couple year protection) per 100 MWRA. The more dispensing of FP methods, especially IUDs and implants (which have a high conversion factor), the greater the value of this indicator. This indicator includes methods dispensed to current users and new users. In other words, and according to the annual FP report, this indicator reflects the percent of MWRA protected from pregnancy for 1 year.

For the development of policy briefs at the central, governorate, and district levels, the “title” should be added and phrased as a suggested recommendation, as, for example, demand creation through training of community members is pivotal to increasing FP new users in Souhag Governorate. The “Introduction” section includes topic importance, objectives, methodology, sources of data, and who conducted data management. The “Major Results” section includes specific results that guide recommendations. The “Recommendation” section should be derived from the key findings.

### Strengths of the study

The study presented collaboration between MOHP and the Public Health Department at Cairo University. The topic focuses on the FP services provided to 15799805 MWRA throughout Egypt via 6038 MOHP FP clinics. Working on all service statistics data covering 1 year prevents sampling errors. Functioning on the current MIS-FP report and developing adjusted record and data analysis to provide a valuable report with KPI without requiring more data or extra efforts from MIS could make the MOHP-FP sector accept the suggested reports developed by the researchers. The designed report contains useful information for developing policy briefs, tracking indicators over time, and communicating information feedback between governorates and the central level. The final report developed by the researcher is presented as two tables and four colored graphs, with no narrative or comments. This is essential for the MOHP-FP sector to describe, elaborate, and suggest their practical recommendations. The current study has implications for the MOHP-FP Monitoring and Evaluation system’s ability to use such a KPI. The researchers’ suggested report contents support needs assessment activities by involving the MOHP contraceptive security department and logistics management department in order to set action plans for supplying specific profiles for method mixes of FP methods at different times. The study is an institutional-based study, i.e., MOHP. The same concept of using KPI for monitoring and evaluation could be applied to different organizations irrespective of their missions, e.g., those working in education, health, economic activities and others. Ranking governorates according to the values of the KPIs and displaying them in colored info-graphs using traffic signal colors makes them self-explanatory, clear, and easy to understand by policymakers at all levels. Ranking of governorates according to KPIs helps MOHP staff at all levels to set their action plans to have a good position in the ranking info-graphs.

### **Limitations of the study**

The study results could not be generalized. However, the methodology could be generalized at national and international levels to maximize the use of KPI through MIS . The use of service statistics hard copies makes it difficult to conduct cross tabulations. The use of data that dated back to 2014 was imposed by the MOHP that banned the use of hard and soft copies of the MIS reports after 2014.

## Conclusion

The MIS-MOHP-FP sector service statistics quarterly and annual reports could be adjusted through the management of data to provide key performance indicators for tracking FP services overtime and across governorates. Organizing the governorates according to their distribution across Egypt's regions and fixing the position of each governorate in the MIS reports would facilitate tracking of FP services for each governorate at central and governorate level. The reason for the setting of the variables in the formats of data collection as well as the electronic reports allowed a reduction of the variables from 25 to 15 variables. The use of the 15 key performance indicators developed from such variables could adjust MIS reports to track the performance of MOHP-FP clinics’ services overtime. Those indicators could be used in ranking governorates from the best to the worst performance groups. The FP-MIS final report could be presented in three pages. Page 1 presents a table that includes governorates by eight indicators (age, parity, and the six contraceptive methods currently used). Page 2 presents a table that includes the governorate by seven indicators (first visit, new clients, 4 reasons for the visit, and CCR). Page 3 includes four colored charts that present the rank order of the governorates, from best to least favorable. Additionally, adjustment of the FP-MIS report to include analyzed data and indicators rather than counts and numbers could facilitate considering FP information by policy makers, programmers, and implementers of services at all levels. Consequently, the MIS-FP service statistics report could guide decision-making regarding improving the supply and demand sides of FP services at all levels.

## Data Availability

The datasets used and/or analyzed during this study are available from the corresponding author on reasonable request.

## Abbreviations

CCR: Contraceptive coverage rate
FP: Family planning
FPS: Family planning sector
KPI: Key performance indicators
IUD: Intrauterine device
MIS: Management information system
MOHP: Ministry of Health and Population (MOHP)
MWRA: Married women in the reproductive age
OC: Oral contraceptives

## References

[1] Abdel Aziz SH. UNFPA Egypt country office. In: Trends of fertility levels in Egypt in recent years by. 2019. https://egypt.unfpa.org/sites/default/files/pub-pdf/. Accessed Feb 2021.

[2] Ministry of Health and Population [Egypt], El-Zanaty and Associates [Egypt], and ICF International (2015). Egypt Demographic and Health Survey 2014.

[3] Chalow J, Chola L, McGee S, Tugendhaft A, Pattinson R (2015). Kate Kerber et al. Triple return on investment: the cost and impact of 13 interventions that could prevent stillbirths and save the lives of mothers and babies in South Africa. BMC Pregnancy Childbirth..

[4] Ministry of Health and Population (MOHP) (2002). National Population Policy and Strategies (2001-2017).

[5] National Population Council (2015). Quarterly statistical report on family planning services (July – September 2015). (In Arabic).

[6] Abdel-Razik MSA (2013). Reasons of shift from IUD to oral contraceptives among MOHP clients in Menofia governorate 2007-2012 report, MOHP and UNFPA.

[7] Khalifa M, Abdelaziz W, Sakr E. Changes in contraceptive use dynamics in Egypt: Analysis of the 2008 and 2014 Demographic and Health Surveys. DHS working papers No. 132. Rockville: ICF; 2017. https://dhsprogram.com/pubs/pdf/WP132/WP132.pdf. Accessed 4 Apr 2021.

[8] Leite IC, Gupta N. Assessing regional differences in contraceptive discontinuation, failure and switching in Brazil. Reprod Health. 2007;1:1–10. 10.1186/1742-4755-4-6. 10.1186/1742-4755-4-6 PMC1976606 17623076

[9] D'Antona Ade O, Chelekis JA, D'Antona MF, Siqueira AD (2009). Contraceptive discontinuation and non-use in Santarém. Brazilian Amazon. Cad Saude Publica..

[10] Egypt National Population Council (NPC)- Annual statistical report (2005 - 2010).

[11] Khalifa M, Abdelaziz W, Metwally S, Sakr E (2020). The recent increase in contraceptive discontinuation in Egypt. J. Biosoc. Sci..

[12] Magnani RJ, Ross J, Weinberger M. Can family planning service statistics be used to track population-level outcomes? 2018;6(1):93–102. 10.9745/GHSP-D-17-00341. 10.9745/GHSP-D-17-00341 PMC5878083 29467167

[13] Abdel-Razik MSA, Ibrahim H (2008). Reasons for decrease in contraceptive coverage rate (CCR) in Egypt 2002 -2007 National Population Council.

[14] Programming strategies for postpartum family planning. Available from: https://apps.who.int/iris/bitstream/handle/10665/93680/9789241506496_eng.pdf. Accessed 24 Apr 2021.

[15] Using contraceptive security: a toolkit for policy audiences. Available from https://www.prb.org/resources/contraceptivesecurity-a-toolkit-for-policy-audiences/. Accessed 21 May 2021.

[16] Adongo PB, Tapsoba P, Phillips JF, Tabong PTN, Stone A, Kuffour E, et al. The role of community-based health planning and services strategy in involving males in the provision of family planning services: a qualitative study in Southern Ghana. Reprod Health. 2013;10:36. 10.1186/1742-4755-10-36. 10.1186/1742-4755-10-36 PMC3726500 23890362

[17] Lavis JN, Permanand G, Oxman AD, Lewin S, Fretheim A. SUPPORT Tools for evidence-informed health Policymaking (STP) 13: preparing and using policy briefs to support evidence-informed policymaking. Health Res Policy Sys. 2009;7(13). 10.1186/1478-4505-7-S1-S13. 10.1186/1478-4505-7-S1-S13 PMC3271824 20018103

